# The effectiveness of extra corporeal shock wave therapy for plantar heel pain: a systematic review and meta-analysis

**DOI:** 10.1186/1471-2474-6-19

**Published:** 2005-04-22

**Authors:** Colin E Thomson, Fay Crawford, Gordon D Murray

**Affiliations:** 1 School of Health Sciences, Queen Margaret University College, Edinburgh, EH6 8HF, UK; 2 Tayside Centre for General Practice, The University of Dundee, The Mackenzie Building, Kirsty Semple Way, Dundee, DD1 4BF, UK; 3 Public Health Sciences, The University of Edinburgh Medical School, Teviot Place, Edinburgh, EH8 9AG, UK

## Abstract

**Background:**

There is considerable controversy regarding the effectiveness of extracorporeal shock wave therapy in the management of plantar heel pain. Our aim was to conduct a systematic review of randomised controlled trials to investigate the effectiveness of extracorporeal shock wave therapy and to produce a precise estimate of the likely benefits of this therapy.

**Methods:**

We conducted a systematic review of all randomised controlled trials (RCTs) identified from the Cochrane Controlled trials register, MEDLINE, EMBASE and CINAHL from 1966 until September 2004. We included randomised trials which evaluated extracorporeal shock wave therapy used to treat plantar heel pain. Trials comparing extra corporeal shock wave therapy with placebo or different doses of extra corporeal shock wave therapy were considered for inclusion in the review. We independently applied the inclusion and exclusion criteria to each identified randomised controlled trial, extracted data and assessed the methodological quality of each trial.

**Results:**

Six RCTs (n = 897) permitted a pooled estimate of effectiveness based on pain scores collected using 10 cm visual analogue scales for morning pain. The estimated weighted mean difference was 0.42 (95% confidence interval 0.02 to 0.83) representing less than 0.5 cm on a visual analogue scale. There was no evidence of heterogeneity and a fixed effects model was used.

**Conclusion:**

A meta-analysis of data from six randomised-controlled trials that included a total of 897 patients was statistically significant in favour of extracorporeal shock wave therapy for the treatment of plantar heel pain but the effect size was very small. A sensitivity analysis including only high quality trials did not detect a statistically significant effect.

## Background

Plantar heel pain (plantar fasciitis) can be debilitating, often with severe limitations on activity. Typically, patients present with pain in the plantar aspect of the heel whilst walking, particularly after rest. Pain on first weight-bearing in the morning is a prominent diagnostic feature. The precise nature of the condition is poorly understood but literature suggests it is an enthesitis at the attachment of the plantar fascia to the plantar medial tubercle of the calcaneum.

A systematic review of the management of heel pain has highlighted the paucity of evidence for managing the condition. The review concluded that treatments used to reduce heel pain, including steroid injections, NSAIDs, night splints, orthoses and stretching regimes, seem to bring only marginal gains [1]. Extracorporeal shock wave therapy (ESWT) was originally used for lithotripsy, but within the last 10 years has become increasingly used to treat musculoskeletal injuries including calcific tendinitis of the shoulder [2], lateral epicondylitis (tennis elbow) [3-5], non-union or delayed osseous union [6] and plantar heel pain [1,7].

Non-systematic review articles, specific to the effectiveness of ESWT in the treatment of plantar heel pain, produce conflicting conclusions. One 'biometric' review [7] suggested that there is insufficient evidence on which to draw conclusions on the effectiveness of EWST and that more trials are required to detect any benefits from the intervention. Bodekker et al [7] incorporated all levels of evidence, including 4 randomised trials, that did not permit pooling of data or statistical synthesis. Study characteristics and quality assessments were provided in the form of lists. Ogden et al's review of ESWT [8] used a "vote counting" method to conclude that ESWT was a useful treatment for plantar heel pain. No quality assessment of the included trials was presented, but a quantitative data synthesis claims success rates ranging from 34% to 88%. Unfortunately, these estimates are not clearly attributed to any specific outcome. Heller and Niethard [9] identified poor trial methodological quality as a barrier to an assessment of the effectiveness of ESWT and were unable to demonstrate any benefit from the treatment in this narrative review article.

There is considerable controversy emerging regarding the use of ESWT for plantar heel pain. Three recent randomised controlled trials have failed to demonstrate a beneficial effect from the use of ESWT [10-12] and it has been suggested that no more clinical trials should be conducted to evaluate this therapy as a treatment for the painful heel [11]. A narrative review article [13] concluded that the available data do not provide substantive support for its use but this prompted correspondence which illustrates the defense for this electrophysical modality in the management of heel pain [14,15]

The purpose of this systematic review was to conduct a rigorous evaluation using a quantitative synthesis of evidence from randomised controlled trials to make a precise estimate of the effectiveness of ESWT. Our aim was to determine if ESWT is effective in the treatment of patients with plantar heel pain when compared with a control group.

## Methods

### Search strategy

Randomised controlled trials were identified by searching the following data sources: The Cochrane Musculoskeletal Injuries Group specialized register of trials (August 2003), the Cochrane Central Register of Controlled Trials (The Cochrane Library issue 3, 2003), MEDLINE (from 1966 to September 2004), EMBASE (from 1982 to September 2004), CINAHL (from 1982 to September 2004) and reference lists of articles and dissertations. In Medline (SilverPlatter), the first two levels of the optimum search strategy [16] were combined with the following subject-specific search terms:

1. HEEL* and SYNDROME*

2. (JOG* or TENNIS* or POLICE* or GONORREAL) near HEEL*

3. PLANTAR near FASCI*

4. explode "FASCIITIS"/ all subheadings

5. (PLANTAR or HEEL* or CALCAN* or FOOT*) near PAIN*

6. HEEL near SPUR

7. "CALCANEUS"/ all subheadings

8. #1 or #2 or #3 or#4 or #5 or #6 or #7

Further details of the search strategy and details of the hand search have been previously published [1], [see Additional file 1].

### Study selection

We considered all randomised controlled trials of plantar heel pain treatments for inclusion in the review. Trials comparing ESWT with placebo or different doses of ESWT were considered. Participants with a clinically confirmed diagnosis of plantar heel pain were included. Adult participants in any trial whether they were part of the general population, athletes, or individuals with seronegative arthropathies and enthesopathies were also considered for inclusion. Any age group was admissible. It was our intention that trials involving children alone, or dealing specifically with young athletes, would be analysed separately. We excluded trials evaluating treatments for plantar heel pain arising from calcaneal fractures, calcaneal tumours, previous surgery for plantar heel pain, or posterior heel pain.

### Outcome measures

We chose morning pain as our *a priori *primary outcome measure for this systematic review. We consider it to be the most important outcome as it is the single most consistent feature of plantar heel pain. Morning pain (pain on first rising, first step pain or start up pain) is universally reported by patients complaining of plantar heel pain and it is also strongly diagnostic for the condition[17]. The secondary outcome measures were walking pain, pressure pain, any measure of disability, quality of life measures and adverse events.

### Data abstraction

Two of the authors (CT,FC) independently applied the inclusion and exclusion criteria to each trial and then extracted data regarding details of the patients (number, mean age and age range, inclusion and exclusion criteria), details of the interventions, nature and timing of outcome measures. Disagreements were resolved by discussion of the articles by the reviewers. We wrote to trialists for additional information on trial methodology (method of randomization) and results (usually requests for data not presented in the original reports such as standard deviations or some other measure of variance).

### Validity assessment

A quality assessment tool[18] adapted for use in a related systematic review of interventions for the treatment of plantar heel pain for the Cochrane Library [1] was applied to each of the included trials. This addressed the following questions:

1. Was the generation of randomization sequence described?

2. Was the method of allocation concealment described?

3. Was an intention to treat analysis used?

We assessed intention to treat on the basis of whether patients were analyzed according to the allocated treatment irrespective of whether this treatment was delivered or not.

4. What number of patients were lost to follow-up?

In assessing loss to follow-up we considered whether authors had presented numbers lost and timing, and the reasons for the loss. We presented the numbers lost to follow up as percentages.

5. Was the outcome assessment blind?

6. Was the patient blind to treatment allocation?

This led to each trial being attributed a quality score out of a maximum of 6 points (Table 1.).

**Table 1:** Quality assessment of included trials

**Author**	**randomisation sequence**	**allocation concealment**	**Assessor blind**	**Patient blind**	**Loss to follow up at trial end**	**Intention to treat**	**Quality score**
Abt et al [21]	No	No	Yes	Yes	11%	No	3
Buch et al [27]	Yes	Yes	Yes	Yes	4%	No	5
Buchbinder et al [10]	Yes	No	Yes	Yes	6%	Yes	5
Cosentino et al [33]	No	No	Yes	Not stated	Not stated	No	1
Haake et al [11]	Yes	Yes	Yes	Yes	16%	Yes	6
Krischek et al [22]	No	No	No	Not stated	6%	No	1
Ogden et al [28]	No	No	Yes	Yes	1.5%	No	3
Rompe et al [32]	No	No	Yes	Yes	16%	No	3
Rompe et al [30]	No	No	No	Yes	Not stated	No	1
Rompe et al [31]	No	No	Yes	Yes	20%	No	3
Speed et al [12]	No	No	Yes	Yes	14%	Yes	4

### Quantitative data synthesis

When measures of variance were not available from the original report, it was our intention to derive these from p-values. When data were available for a pooled estimate of the impact of intervention it was intended that meta-analyses would be conducted for direct comparisons. We intended to present weighted mean differences and 95% confidence intervals for outcomes for each randomised controlled trial and group them in relevant sub-groups according to the specific question they addressed. We intended to use a fixed effects model to estimate the pooled effect as our primary analysis where no evidence of heterogeneity was detected [19]. However, if evidence of heterogeneity was found to be present we intended to use a random effects model [20]. Meta-analyses were generated using *RevMan *software. We planned to perform subgroup analyses and sensitivity analyses, regarding any anomalies with the included trials, methodological scores and industry sponsorship. We proposed to perform a funnel plot to detect publication bias.

## Results

### Selection of trials

**Figure 1:**
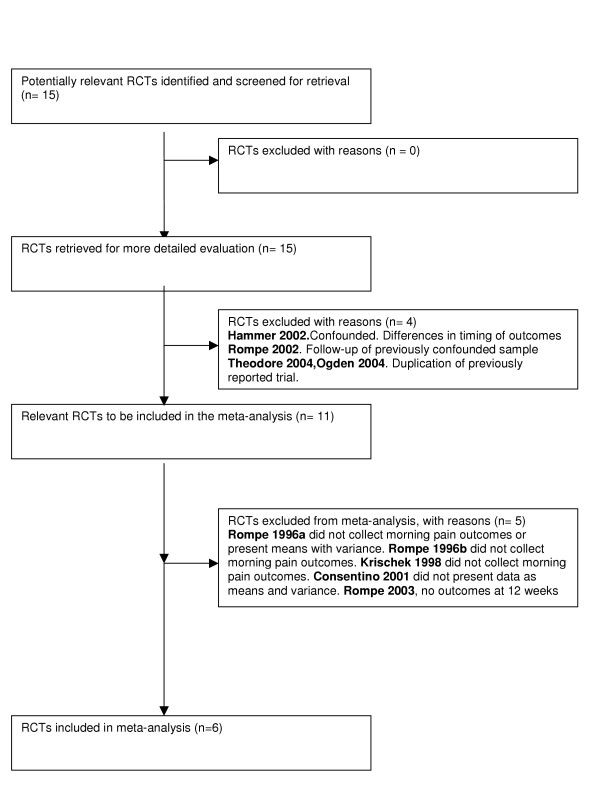
Progress through the stages of the meta-analysis [29].

The search strategy identified a total of 205 studies, of which 15 were identified as RCTs that evaluated ESWT for plantar heel pain. Two of these were translated from German into English [21,22]. Four trials [23-26] were excluded from the review: in one, the intervention and control groups were treated at different time points making valid comparisons of patient outcomes in both groups impossible [24]. The second trial contained five year follow-up data from an RCT published in 1996 [23]. These trial data were confounded by placebo patients receiving additional therapies after 12 weeks. The third [25] and fourth [26]excluded trials were duplicated data previously reported by Buch [27] and by Ogden [28]respectively. The flow diagram in Figure 1 provides details of the included and excluded trials and those included in the final meta-analysis[29].

### Description of included studies

Eleven RCTs were included in this review and they reported data published between 1996–2003 from trials involving 1290 patients [10-12,21,22,27,28,30-33]. Table 1 shows the quality assessment scores and Table 2 and Table 3 the baseline data. The trials evaluated different doses of ESWT against either a placebo dose or a control dose so low as to be considered therapeutically ineffective [10] (Table 4). Only five of the trial reports contained summary statistics to permit pooling of data collected at 12 weeks in a forest plot [10-12,27,28]. Standard deviations were derived from the p value reported in one manuscript in order to incorporate a sixth trial in the meta-analysis, the timing of the outcomes varied between 17 and 20 weeks for this trial [21].

**Table 2:** Baseline characteristics of participants in respective trials. (N/a- data not available).

Author	Age mean (SD and/or range) years		Female:male (% female)		Mean BMI (SD)	
	Treatment group	Control group	Treatment group	Control group	Treatment group	Control group

Abt et al [21]	56.5	57.4	11:6 (64.7)	9:6 (60)	30.1	28.5
Buch et al [27]	50.4 (10.3, 26–69)	53.0 (9.7, 31–72)	61:14 (81.3)	46:26 (63.9)	28.9	28.5
Buchbinder et al [10]	52.2 (12.8)	54.2 (12.0)	46:3 (57.5)	47:3 (58.0)	29.5	28.9
Cosentino et al [33]	55.6 (45–68)		18:12 (60.0)	25:5 (83.3)	N/a	N/a
Haake et al [11]	53.1 (10.8)	52.9 (10.8)	98:37 (72.6)	106:30 (77.9)	29.4 (4.9)	29.7 (4.8)
Krischek et al [22]	54.0	55.0	(56.0)	(72.0)	N/a	N/a
Ogden et al [28]	49.6 (20–79)		171 (65.9)		N/a	N/a
Rompe et al [32]	44.0 (26–61)	49.0 (31–63)	21:29 (42.0)	20:30 (40.)	N/a	N/a
Rompe et al [30]	47.0 (26–61)	51.0 (31–58)	5:10 (33.3)	6:9 (40)	N/a	N/a
Rompe et al [31]	43.0 (32–59)	40.0 (30–61)	10:12 (45.5)	13:10 (56.5)	N/a	N/a
Speed et al [12]	51.7 (25–76)	52.5 (30–73)	26:20 (56.5)	25:17 (59.5)	N/a	N/a

**Table 3:** Baseline characteristics of participants in respective trials continued (N/a- data not available).

Author	Duration of heel pain Median (SD and/or range) months		Base line morning pain VAS score (SD)	
	Treatment group	Control group	Treatment Group	Control group

Abt et al [21]	19.0	19.0	5.7	5.3
Buch et al [27]	20.7 (21.1, 6–120)	24.0 (21.1,6–99)	7.7 (1.4)	7.7 (1.5)
Buchbinder et al [10]	9.0 (2–150)	10.8 (2–222.5)	7.3 (2.5)	6.8 (3.2)
Cosentino et al [33]	8.2(6–12)	8.2(1.2)	N/a	N/a
Haake et al [11]	13.0 (10–24)	13.0 (9–24)	7.8 (2.4)	7.7 (2.3)
Krischek et al [22]	22.0	23.0	N/a	N/a
Ogden et al [28]	32.2	35.9	8.1	8.2
Rompe et al [32]	8.0 (6–19)	10.0 (6–20)	N/a	N/a
Rompe et al [30]	16.0 (12–36)	22.0 (12–38)	N/a	N/a
Rompe et al [31]	20.0 (12–60)	18.0 (12–72)	6.9 (1.3)	7.0 (1.3)
Speed et al [12]	16.7 (12–312)	13.5 (12–312)	7.4(2.0)	7.0(2.0)

**Table 4:** Details of studies included in the systematic review

Author	Included in meta-analysis	Local anesthetic to both groups	Details of placebo/sub therapeutic dose	Ultrasound guidance	N	Weighted mean difference – morning pain (95%CI)	Timing of outcomes (weeks)
Abt et al [21]	yes	yes	Absorbent block	Not stated	32	2.00 (0.47 to 3.53)	19,32
Buch et al [27]	yes	yes	Absorbent foil	yes	150	0.70 (-0.26 to 1.66)	12
Buchbinder et al [10]	yes	no	6000 to 7500 vs 300 impulses	yes	178	-0.50 (-1.55 to 0.55)	6,12
Cosentino et al [33]	no	no	Not stated	yes	60	Not available	4,12
Haake et al [11]	yes	yes	Polythene foil barrier	Not stated	272	0.50 (-0.31 to 1.31)	6,12, 52
Krischek et al [22]	no	no	1500 vs 300 impulses	Not stated	50	Not available	6,12
Ogden et al [28]	yes	yes*	Styrofoam block	no	260	0.56 (-0.26 to 1.38)	4,8,12
Rompe et al [32]	no	no	3000 vs 30 impulses	Not stated	119	Not available	12, 52
Rompe et al [30]	no	no	1 cm gap	no	36	Not available	3,6,12,24
Rompe et al [31]	no	no	Reflecting pad	yes	45	2.60 (1.37 to 3.83)	26,52
Speed et al [12]	yes	no	Focus outside patient	yes	88	-0.36 (-1.66 to 0.94)	4,8,12,24

* Local anaesthetic was used for both groups but was different for the placebo group and the treatment group.

Table 2 and table 3 present details of the baseline pain scores, and demographic variables for participants from all eleven included trials. All included adult patients only. The duration of pain was greater than 6 months in ten trials [11,12,21,22,27,28,30-33]. In one trial [10] the duration of pain was shorter than six months for some patients but no patient had a duration of pain less than 8 weeks. The duration of pain ranged from 8–600 weeks and 8–980 weeks for the ESWT and placebo groups respectively. The median values for duration of pain were 36 weeks and 43 weeks. The demography of the patients in this systematic review of ESWT for plantar heel pain was similar to those patients who have participated in evaluations of other interventions for heel pain [1]. The effects of ESWT in people who had a calcaneal spur on x-ray [4,32], were running athletes [31], were being considered for surgical intervention [30,32,32], had failed to respond to conservative treatments [27,28,30,32], or were defined as recalcitrant cases [22], were all included in this systematic review.

There was diversity in the types of primary and secondary outcomes collected from patients in the 11 RCTs. Table 5. summarizes the most commonly reported outcomes measures indicating, where available, the outcomes provided. With the exception of three trials [22,30,32] all presented data for visual analogue scale scores of morning pain. Walking pain is a relevant outcome measure and was reported by eight trials [10,11,21,22,30,32,33]. Only two of these trials contained compatible data [30,32] and insufficient data are provided to permit pooling. The remaining trials described a wide variety of walking ability using incongruous scoring systems. Six of the trials [11,21,22,30,32,33], show a favourable outcome for walking pain after ESWT. Resting and night pain are not common symptoms of heel pain, in our experience, but data for these outcomes were collected in four trials [12,21,30,32]. Five trials reported the collection of pressure pain outcomes from the application of pressure from either a manual application or an electronic device [21,27,28,30,32]. Other outcomes reported were Roles and Maudsley scores [11,21,27], Maryland Foot score[10], SF12 [27], SF36 [10], problem elicitation technique [10] and The Ankle Hindfoot Scale [31].

**Table 5:** Summary of most commonly reported outcomes measures at 12 weeks (or nearest point to). P values relate to active treatment versus placebo or reduced dose. * Indicates a statistical significant difference in favour of EWST treatment. Figures in parentheses are 95% confidence intervals. Where p-values were not provided, the values for mean and standard deviations [SD] are given, I indicates EWST group, II indicates placebo group. "Favours ESWT" indicates a better outcome for ESWT where neither of the previous details are provided.

**Author**	**Morning/start up pain**	**Overall pain**	**Walking ability /activity related**	**Foot specific score**	**Pain at rest (100 mm VAS)**	**Pain on pressure**	**Night pain/evening pain**	**End point**
Abt et al [21]	P = 0.016*	-	Favours ESWT	-	P = 0.01*	P = 0.26	P = 0.01*	**19 weeks**
Buch et al [27]	P = 0.0309	-	P = 0.7377	I. (49.1–71.5) II.(32.9–55.9) AOFAS	-	Not significant	P < 0.4338	**12 weeks**
Buchbinder et al [10]	P = 0.92 (-12.7 – 13.1)	P = 0.99 (-10.3–11.5)	P = 0.0.72 (0.6–1.9)	P = 0.85 (-7.6–5.3) Maryland FS	-	-	-	**12 weeks**
Cosentino et al [33]	P < 0.0001*	-	P < 0.0001*	-	P < 0.0001*	-	-	**12 weeks**
Haake et al [11]	I mean = 4.0, SD = 3.2 II mean = 4.5, SD = 3.0	-	Favours ESWT	-	I mean = 2.4, SD = 2.6 II mean = 2.4, SD = 2.5	I mean = 4.0, SD = 3.2 II mean = 4.3, SD = 3.2	I mean = 1.5, SD = 2.4 II mean = 1.8, SD = 2.5	**12 weeks**
Krischek et al [22]	-	Favours ESWT	Favours ESWT	-	-	Favours ESWT	-	**12 weeks**
Ogden et al [28]	Favours ESWT	-	-	-	-	Favours ESWT	-	**12 weeks**
Rompe et al [32]	-	-	Favours ESWT	-	P < 0.0001*	P < 0.0001*	P < 0.0001*	**12 weeks**
Rompe et al [30]	Favours ESWT	Favours ESWT	P < 0.0001*	-	P < 0.05*	P < 0.0001*	P < 0.05*	**12 weeks**
Rompe et al [31]	P = 0.0004*	-	-	P = 0.0025* AOFAS	-	-	-	**26 weeks**
Speed et al	P = 0.664 (0.656–1.271)	P = 0.246 (0.626–1.093)	-	-	-	-	P = 0.378 (0.620–1.166)	**12 weeks**

Of the 11 RCTs that met our inclusion criteria, eight were placebo controlled trials [11,12,21,27,28,31-33]. Three trials used a low, sub-therapeutic dose as control [10,22,30]. The doses for the intervention groups and methods used to disable the equipment for the placebo group and the sub-therapeutic groups are provided in Table 2 and Table 3. The dose of ESWT varied between trials in both energy levels and the number of impulses administered. With the exception of two trials, [10,12], all excluded patients had the condition for less than six months. Only one trial [10] did not require patients to have exhausted conservative therapies for recalcitrant plantar heel pain before embarking on treatment with ESWT but information presented reveals that the majority of patients did receive a number of conservative therapies. Krischek et al [22] and Rompe et al [31] included only patients whose next management option was surgery.

### Quantitative data synthesis

**Figure 2:**
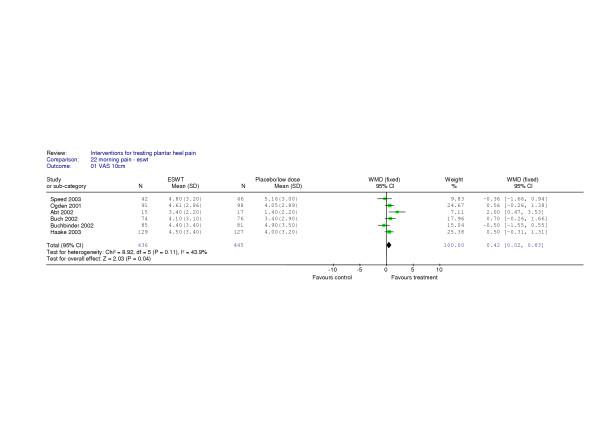
Pooled estimates of 10 cm VAS scores for morning pain at 12 weeks

Figure 2. shows the pooled analysis of data from 6 trials which produce a weighted mean difference of 0.42 in favour of ESWT. This treatment effect is statistically significant (p = 0.04), but the effect is small (95% confidence interval of 0.02 to 0.83) with respect to morning pain (first step pain). All outcomes were taken at 12 weeks, except for one trial [21] which reported the first outcome measured at (on average) 19 weeks. There was no evidence of heterogeneity (p = 0.11) and a fixed effects model was used.

We repeated the meta-analysis excluding the data from the trial by Abt et al [21], the only trial for which we had to impute measures of variance. The resultant weighted mean difference was 0.30 in favour of ESWT, with a 95% confidence interval of -0.12 to 0.72. This effect is no longer statistically significant.

We performed a sensitivity analysis for the quality of trial reports by dividing the six trials into two groups; those that received a quality assessment score of four or more [10-12,27] and those receiving a score of less than four [21,28] to perform meta-analyses using fixed effects models. The four better quality trials produced a non significant result (weighted mean difference 0.21, 95% confidence interval -0.29 to 0.70 cm, p = 0.41) whereas the two trials scoring less than three produced a significant result in favour of active treatment (weighted mean difference -0.90, 95% confidence interval -1.62 to -0.19, p = 0.01).

### Adverse events

Two trials did not report adverse events [12,30]. Buchbinder et al [10] reported pain for one week by one patient in each arm of the trial; one patient in the active arm of the trial reported a sensation of heat and numbness, whilst another complained of bruising. One patient in the placebo arm complained of a burning sensation in the heel and ankle. Ogden et al [28] reported 38 procedure related complications, 18 of which occurred in the active treatment arm. The most common procedure related complications were mild neurological symptoms (numbness, tingling). One patient who suffered a plantar fascial rupture 4 weeks after active treatment had undergone multiple cortisone injections prior to embarking upon treatment with ESWT.

Haake et al [11] reported a statistically significant difference in the number of side effects in the active and placebo groups; OR 2.26 (95% confidence interval 1.02 to 5.18) [11]. These were; skin reddening, pain and local swelling. The same authors [11] also describe less frequent complaints of dizziness, sleep disturbance haematoma, nausea and hair loss as non-serious effects and discounted one report of a deep vein thrombosis in a placebo participant as a co-incidental event. In two trials, [31,32] the unpleasant nature of ESWT experienced by patients during treatment was reported. These sensations were regarded as less unpleasant than local cortisone infiltration. Krischek et al [22] reported that there were no adverse events noted in trial participants.

### Industry sponsorship

Companies who produce ESWT equipment provided some sponsorship in three trials [11,27,28] (Table 6). One trial [28] was the basis for the first Food and Drug Administration (FDA) approval for ESWT. A financial interest with HealthTronics was declared in correspondence following the publication of the trial [34,35]. The trial by Buch et al [27] was sponsored by Dornier Med tech Inc and the data were also used to gain approval for the use of ESWT in the management of plantar heel pain from the FDA. Haake et al [11] stated no competing interests but did declare that a manufacturer of ESWT equipment had provided the machine used in the trial. Two trials [10,12] declared funding from sources other than industry. In the remaining trials there was no explicit declaration of competing interests [21,22,30-33] (Table 6).

**Table 6:** Details of ESWT devices, dose of impulses administered.

Author	Device	ESWT impulse dose × number of treatments	Low energy/ high energy (energy level)	Details of sponsorship
Abt et al [21]	Ossatron High Medical Technology	1000 × 2	Low Energy (0.08 mJ/mm^2^	No declaration
Buch et al [27]	Epos Ultra Dornier Medical Systems	3800 total	High energy (0.03–0.36 mJ/mm^2 ^-total 1300 mJ/mm^2^)	Industry sponsored trial but this was not declared
Buchbinder et al [10]	Epos Ultra Dornier Medical Systems	2000–2500 × 3	Low energy (0.02–0.33 mJ/mm^2^-total 1000 mJ/mm^2^)	Declared funding – not from industry
Cosentino et al [33]	Orthima Direx Med Sys Ltd	1200 × 6	Not stated (0.03–0.4 mJ/mm^2^)	No declaration
Haake et al [11]	Epos Ultra Dornier Medical Systems	4000 × 3	Low energy (0.08 mJ/mm^2^-total 0.96 J/mm^2^)	Declared: industry provided machine
Krischek et al [22]	Osteostar Siemans	500 × 3	Low energy (0.08 mJ/mm^2^)	No declaration
Ogden et al [28]	Ossatron High Medical Technology	1500 total	High energy (0.22 mJ/mm^2^-total 324.25 J)	Industry sponsored trial but this was not declared
Rompe et al [32]	Osteostar Siemans	1000 × 3	Low energy (0.06 mJ/mm^2^)	No declaration
Rompe et al [30]	Osteostar Siemans	1000 × 3	Low energy (0.06 mJ/mm^2^)	No declaration
Rompe et al [31]	Sonocur Plus Siemens	2100 × 3	Low energy (0.16 mJ/mm^2^)	No declaration
Speed et al [12]	Sonocur Plus Siemens	1500 × 3	Low energy (0.06 mJ/mm^2^)	Declared funding – not from industry

## Discussion

The lack of convergence of findings from randomised evaluations of EWST for plantar heel pain has resulted in clinical uncertainty about its effectiveness. Within this systematic review, we have been able to evaluate the effectiveness of ESWT in a meta-analysis and used the pooled data to arrive at more precise conclusions about its usefulness in clinical practice.

The meta-analysis shows a statistically significant benefit with ESWT on plantar heel pain from outcomes of 897 patients' VAS scores of morning (first-step) pain assessed at or around 12 weeks but we do not consider this clinically significant since the observed benefit equates to less than one half centimeter on a 10 cm VAS. The 95% confidence interval is compatible with a mean treatment benefit of at most 0.83 cm. A sensitivity analysis including only those higher quality trials did not produce evidence of a statistically significant benefit. Only one trial included in the review discussed what might constitute a clinically meaningful reduction in plantar heel pain: Buchbinder et al [10], suggest that 0.7 cm reduction of heel pain may not be clinically relevant.

We included one trial in the meta-analysis which used sub-clinical doses as controls [10] and combined these patient outcomes with those from trials which used sham treatments as controls [11,12,21,27,28]. All six trials [10-12,21,27,28] also used different doses of ESWT but, despite the differences in the use of control interventions and doses, no evidence of heterogeneity in the patient outcomes was detected in the pooled estimate (figure 2). Nor does there appear to be a dose-response relationship for ESWT; trials using both high and low doses have reported similar effects as is evident from the estimates from the trials by Haake et al [11] and Abt et al [21] (Table 6, figure 2).

We were grateful to the authors of trials included in this review who provided supplementary data in response to our correspondence [10,11] but disappointed that data from all 11 trials were not available to us. Five trials were not included in the meta-analysis either because adequate data were not provided [22,33] the timing of the outcomes differed greatly from the other trials [31] or the outcomes were clinically irrelevant [30,32]. Consequently, information about the effects of ESWT in 310 patients with heel pain was effectively lost to re-analysis. Any future reporting of patient outcomes should include means of pain scores with measures of variance in order that new trials can be included in meta-analyses and weighted mean differences and confidence intervals calculated [36].

Rompe et al conducted a small trial (n = 40) which evaluated the benefits of ESWT in running athletes [31] and reported a mean difference of 2.60 (95% confidence interval 1.37 to 3.83) for morning pain at 6 months. This effect size is statistically significantly different from the combined outcomes presented in Figure 2 but not statistically different from the mean difference in outcomes reported in the small trial by Abt et al [21] 2.00 (95%confidence interval 0.47 to 3.53) at 19 weeks (n = 37). That the two smallest trials included in the review should produce between-group comparisons of pain in the morning that reach statistical significance when estimates from larger studies do not is surprising. Sample size is an important factor in experimental bias in clinical trials as effect size estimates from small studies can be highly variable [37]. The effect sizes from these small studies may be due to ESWT being beneficial in certain sub groups within the population (e.g. runners), or may be as a result of a failure to blind the participants successfully to their treatment allocation, as previously reported by one of the authors [30]. Alternatively, these data may be aberrant values that are more likely to occur by chance in small studies than larger ones [38].

ESWT was not considered a suitable therapy for the first-line management of heel pain by the majority of the investigators. This may be because of limited access to this relatively new and expensive equipment or, more likely, because of the favourable natural history of this condition.

In the absence of a validated heel pain specific outcome measure, our *a priori *choice of morning pain as the primary outcome measure was vindicated by eight of the of the eleven included trials collecting morning pain or first step/start up pain outcomes. One trialist [10] used a *problem elicitation technique *which confirmed "walking after getting out of bed in the morning" as the most frequently reported problem by patients with heel pain. We had planned to pool additional secondary outcome measures, such as walking pain, but this was not possible because of the diversity of the outcome measures used and differences in the data collected. Some of the outcomes that have been used to assess the effects of treatments were clinically irrelevant in our opinion [30-33]. Night pain and resting pain are not symptoms that we commonly encounter in patients seeking treatment for plantar heel pain. Three trials [11,21,27] incorporated the Roles Maudsley scale and one trial [10] used the Maryland Foot Score as measures of disability. It is commendable that two of the investigators [10,27] used generic health outcomes, SF36 and SF 12 respectively. Future trials should include outcomes of disability as well as the impact on health related quality of life and not just pain when assessing the effect of interventions for heel pain.

Of the eight outcomes listed in Table 5, only "pain at rest" is distinct with four of the five trials [11,21,30,32,33] favouring ESWT compared with placebo or reduced dose. As previously discussed, this outcome measure is not a key feature of plantar heel pain. All other outcome measures are equivocal.

Minimal side effects were reported by Abt et al [21] and Buchbinder et al [10]. The most frequently reported adverse event from the use of ESWT is pain [11,27,32,33] which appeared to affect some patients both during and after the procedure.

The quality of reporting varied amongst trials. The three most recent trials [10,11,31] all received above average quality scores for trial reporting. This is an encouraging development for those interested in improving the outcomes for patients who have heel pain and may reflect both the use of checklists such as the CONSORT statement [36] for trial reports now demanded by many journal editors as well as a greater awareness of good trial reporting practice by trialists themselves. There was however, a contrast in the results obtained from the four better quality trials, scoring three or above, when meta-analyzed separately from the two poorer quality trials. Better quality trials did not favour ESWT whilst the poorer quality ones did.

### Industry sponsorship

At least two of the trials included in our meta-analysis, received some form of sponsorship from a company manufacturing ESWT [27,28] although this has not been made explicit within the published papers. Both these trials reported significant benefit from ESWT. One further trial Haake et al [11] declared being supplied with the ESWT equipment and reported no statistically significant effects between the two groups. Six of the trials [21,22,30-33] have not made it clear whether there is any conflict of interest or not. In a systematic review to investigate whether the funding of drug studies by the pharmaceutical industry is associated with bias, Lexchin et al [39] concluded that industry sponsorship was more likely to produce results favouring the sponsors' product than studies funded from other sources.

### Publication bias

In view of concerns about publication bias, it is encouraging that three large, negative trials have been published in high impact journals. We were unable to recognize the existence of small, unpublished studies showing no statistically significant benefits. However, the existence of any such trials would only serve to endorse the findings of the meta-analysis in this systematic review.

## Conclusion

It has been suggested that the poor outcomes reported by recent randomised controlled trials evaluating ESWT for plantar heel pain means no further trials should be conducted [11]. A meta-analysis of data from six randomized controlled trials that included a total of 897 patients was statistically significant in favour of extracorporeal shock wave therapy for the treatment of plantar heel pain but the effect size was very small. When the two poorest quality trials, and therefore the greatest source of bias, are removed from the meta-analysis, the result is not statistically significant. This systematic review does not support the use of ESWT for plantar heel pain in clinical practice.

## Abbreviations

ESWT: Extracorporeal shock wave therapy

## Competing interests

The author(s) declare that they have no competing interests.

## Authors' contributions

FC and CT performed the literature search, extracted data, performed data analyses and compiled the manuscript. GM performed data analyses and compiled the manuscript. We can confirm that all authors have access to all data in the study and that they held final responsibility for the decision to submit for publication.

## Pre-publication history

The pre-publication history for this paper can be accessed here:



## Supplementary Material

Additional File 1 "Details of EMBASE and MEDLINE search strategies". this additional file contains full details of the EMBASE and MEDLINE search strategies that were used for this systematic review. Only an abbreviated version was provided within the text. Click here for file
