# Supplementary material for: The effectiveness of extra corporeal shock wave therapy for plantar heel pain: a systematic review and meta-analysis
Source: BMC Musculoskelet Disord. 2005 Apr 22;6:19. doi: 10.1186/1471-2474-6-19 (PMC1097736; doi:10.1186/1471-2474-6-19)
Supplement: Additional File 1 — "Details of EMBASE and MEDLINE search strategies". this additional file contains full details of the EMBASE and MEDLINE search strategies that were used for this systematic review. Only an abbreviated version was provided within the text. [file 1471-2474-6-19-S1.doc]

Database: EMBASE <1996 to 2004 Week 38>

Search Strategy:

--------------------------------------------------------------------------------

1 Heel Pain/ (78)

2 Plantar Fasciitis/ (50)

3 Achilles Enthesopathy/ (1)

4 (heel$ adj6 (syndrom$ or jog$ or tennis$ or police$ or gonorrh$)).tw. (40)

5 (plantar adj3 fasci?tis).tw. (217)

6 (pain$ adj3 (plantar or heel$ or calcan$ or foot$)).tw. (789)

7 (heel adj3 spur).tw. (38)

8 (enthes$ and (heel$ or foot$ or feet or plantar)).tw. (53)

9 or/1-8 (1009)

10 exp Randomized Controlled trial/ (74491)

11 exp Double Blind Procedure/ (34103)

12 exp Single Blind Procedure/ (3976)

13 exp Crossover Procedure/ (11387)

14 Controlled Study/ (1257432)

15 or/10-14 (1264295)

16 ((clinical or controlled or comparative or placebo or prospective$ or randomi#ed) adj3 (trial or study)).tw. (143588)

17 (random$ adj7 (allocat$ or allot$ or assign$ or basis$ or divid$ or order$)).tw. (31045)

18 ((singl$ or doubl$ or trebl$ or tripl$) adj7 (blind$ or mask$)).tw. (33575)

19 (cross?over$ or (cross adj1 over$)).tw. (14117)

20 ((allocat$ or allot$ or assign$ or divid$) adj3 (condition$ or experiment$ or intervention$ or treatment$ or therap$ or control$ or group$)).tw. (45318)

21 or/16-20 (207654)

22 or/15,21 (1343195)

23 limit 22 to human (812756)

24 and/9,23 (296)

25 limit 24 to yr=2002 - 2004 (145)

26 from 25 keep 1-10 (10)

27 from 25 keep 1-10 (10)

Database: Ovid MEDLINE(R) <1966 to September Week 2 2004>

Search Strategy:

--------------------------------------------------------------------------------

1 Heel/ and Pain/ (328)

2 Fasciitis, Plantar/ (64)

3 (heel$ adj6 (syndrom$ or jog$ or tennis$ or police$ or gonorrh$)).tw. (115)

4 (plantar adj3 fasci?tis).tw. (286)

5 (pain$ adj3 (plantar or heel$ or calcan$ or foot$)).tw. (1449)

6 (heel adj3 spur).tw. (86)

7 (enthes$ and (heel$ or foot$ or feet or plantar)).tw. (78)

8 or/1-7 (1834)

9 8 not Infant, Newborn/ (1753)

10 randomized controlled trial.pt. (193912)

11 controlled clinical trial.pt. (67230)

12 Randomized Controlled Trials/ (34253)

13 Random Allocation/ (51876)

14 Double-Blind Method/ (79756)

15 Single-Blind Method/ (8421)

16 or/10-15 (328884)

17 Animals/ not Human/ (2836902)

18 16 not 17 (311465)

19 clinical trial.pt. (391656)

20 exp Clinical Trials/ (158960)

21 (clinic$ adj25 trial$).tw. (103226)

22 ((singl$ or doubl$ or trebl$ or tripl$) adj (mask$ or blind$)).tw. (76300)

23 Placebos/ (23310)

24 placebo$.tw. (86125)

25 random$.tw. (293880)

26 Research Design/ (38922)

27 (latin adj square).tw. (2120)

28 or/19-27 (692851)

29 28 not 17 (642855)

30 29 not 18 (341846)

31 or/18,30 (653311)

32 and/9,31 (178)

33 limit 32 to yr=2002 - 2004 (62)

34 from 33 keep 5-6 (2)

35 from 34 keep 1-2 (2)

36 from 33 keep 1-62 (62)
